# Supplementary material for: Assessing adolescents’ critical health literacy: How is trust in government leadership associated with knowledge of COVID-19?
Source: PLoS One. 2021 Nov 24;16(11):e0259523. doi: 10.1371/journal.pone.0259523 (PMC8612506; doi:10.1371/journal.pone.0259523)
Supplement: S1 File — (DOCX) [file pone.0259523.s001.docx]

**S1 File. Scale Items.**

**Items for News Source**

**Item Stem:** How often do you encounter COVID-19 information from each of the following sources? (Likert-Type Scale; 1= *Never* to 7= *Every day*):

1. Newspapers in print format
2. Newspapers in online format
3. Facebook
4. Twitter
5. Snapchat
6. Instagram
7. TikTok
8. YouTube

**Items for Trust in Source of Covid-19 Information**

**Item Stem:** How much do you trust the information presented by the [information source] about COVID-19? (Likert-Type Scale; 1= *Don’t trust at all* to 7= *Trust completely*).

1. Teachers
2. Government leader
3. Government
4. World Health Organization
5. News Media
6. Social Media

**Items Assessing COVID-19 Health Literacy in STEM-oriented Adolescents with Percentage Correct per Item**

1. What is “COVID-19”?
   1. Correct Answer: The name of the disease caused by virus SARS-CoV-2 (28% correct).
2. Which of the following is true of COVID-19?
   1. Correct Answer: It can be transmitted in droplets that come out of the mouth while talking (85% correct).
3. Which of the following is true of viruses?
   1. Correct Answer: Large outbreaks of them can be prevented if enough people get a vaccine specifically for them (87% correct).
4. Which of the following is true of this graph?


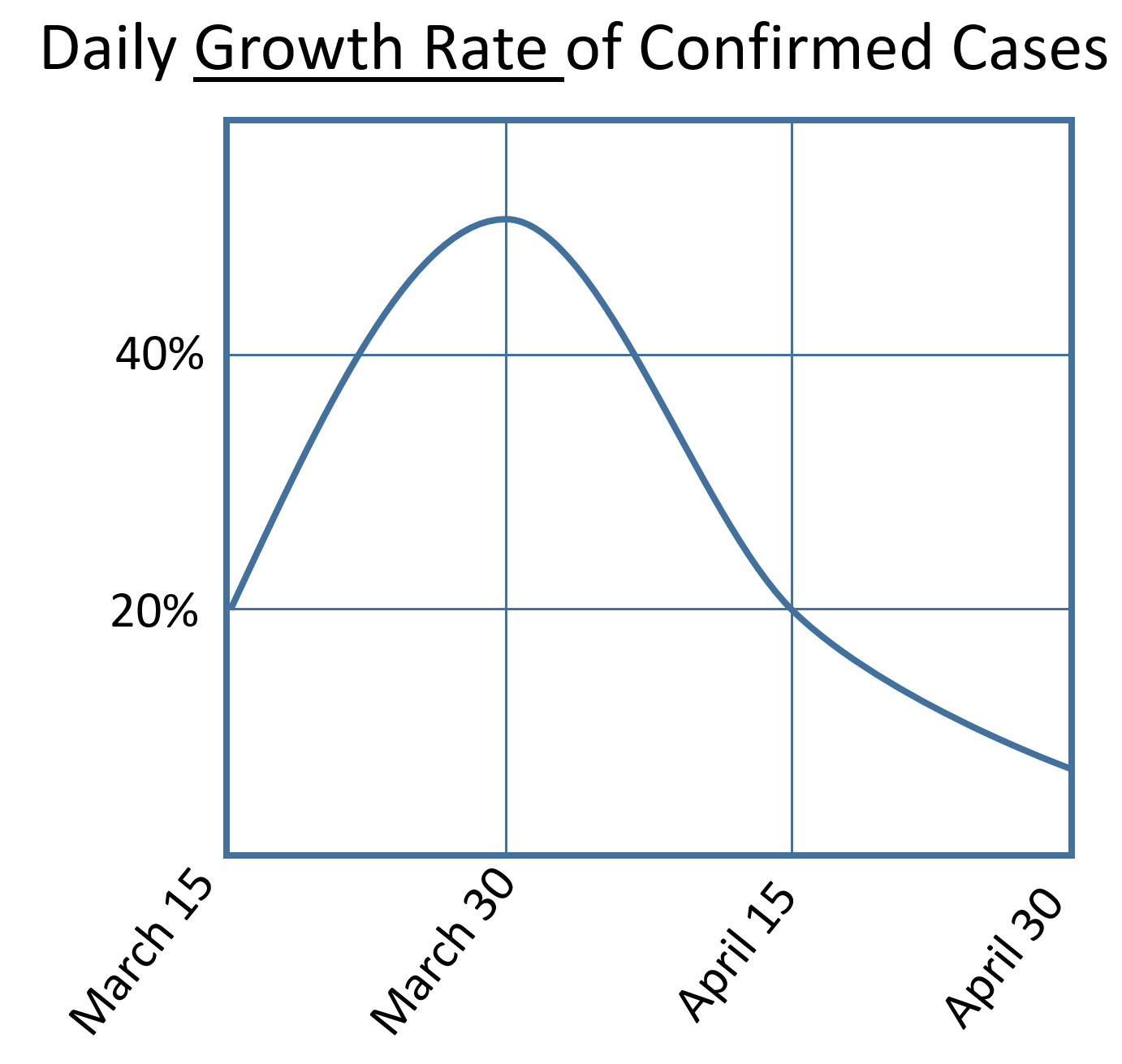


- 1. Correct Answer: This graph shows that on April 30 there were more confirmed cases than on April 29^th^ (22% correct).
