# Supplementary material for: Assessing adolescents’ critical health literacy: How is trust in government leadership associated with knowledge of COVID-19?
Source: PLoS One. 2021 Nov 24;16(11):e0259523. doi: 10.1371/journal.pone.0259523 (PMC8612506; doi:10.1371/journal.pone.0259523)
Supplement: Attachment — Submitted filename: Response to Reviewers.docx [file pone.0259523.s002.docx]

August 31, 2021

PLOS ONE Editorial Board

Dear Dr. Camacho-Rivera,

Thank you for your careful feedback to our manuscript entitled: *Assessing adolescents’ critical health literacy: How is trust in government leadership associated with knowledge of COVID-19?* We appreciate and have addressed your overall comments as well as comments from our reviewer, Dr. Hazmah, throughout our last manuscript draft. In this cover letter, the exact language from the action letter is first quoted in italics, followed by our response in plain text. Minor changes, such as updating existing citations and style requirements, are not detailed here, but are shown in the revised manuscript with tracked changes.

1.) *You indicated that you had ethical approval for your study. In your Methods section, please ensure you have also stated whether you obtained consent from parents or guardians of the minors included in the study or whether the research ethics committee or IRB specifically waived the need for their consent.*

Thank you for this feedback. We clarified in the methods section that we obtained consent from the parents or guardians of minors included in the study from both countries.

2.) *In order to improve reporting, in your methods section, please provide additional information about the participant recruitment method and the demographic details of your participants, and in particular provide additional information about the broader project they were recruited for.*

Thank you for this suggestion. We have included more descriptive information to highlight the international collaboration between the US and UK and described the goal of the original project to assess trajectories adolescents’ science career interests over time.

3.) *In your Data Availability statement, you have not specified where the minimal data set underlying the results described in your manuscript can be found.*

Thank you for pointing out this error. The dataset was previously included in the supplemental files but has now been deposited on the Open Science Framework. It can be found at the following URL: <https://osf.io/nj3bf/>

4.) *How many overall population of STEM students for both countries?*

The overall population of STEM students is 194. There are 105 in the UK and 89 in the US. This information is indicated in the Participants section on pg. 14.

5.) *The authors did mention the limitation of study intern of sampling. The sample size is 194 consisted of 2 categories, does this sample size is enough for hierarchical linear regression?*

Thank you for this question. We included a power analysis using G*Power that is described on page 14 of the manuscript. Power analysis indicated that a sample size of greater than 110 would enable us to detect a small effect.

6.) *What is the effect size of the moderation analysis?*

The effect size of the moderation analysis is indicated by the R^2^ value indicated in Table 3 on page 20 of the revised manuscript. R^2^=.13

7.) *How about the validation of items measurement?*

We added wording to indicate that all the measures were newly developed for this survey. We also included alpha levels to indicate the reliability of applicable measures.

8.) *It would be good if the authors spell out all items measured in the appendix.*

Thank you for this suggestion. We have updated the appendix to include descriptions of all the items measured.

Thank you for your review of our manuscript. We are excited to move forward in the next steps of the revision process.

Sincerely and on behalf of all co-authors,

Channing J. Mathews, PhD

Postdoctoral Research Fellow, Social Development Lab

North Carolina State University
